# Theoretical Analysis of Transcranial Magneto-Acoustical Stimulation with Hodgkin-Huxley Neuron Model

**DOI:** 10.3389/fncom.2016.00035

**Published:** 2016-04-19

**Authors:** Yi Yuan, Yudong Chen, Xiaoli Li

**Affiliations:** ^1^Department of Automation, Institute of Electrical Engineering, Yanshan UniversityQinhuangdao, China; ^2^State Key Laboratory of Cognitive Neuroscience and Learning, IDG/McGovern Institute for Brain Research, Beijing Normal UniversityBeijing, China; ^3^Center for Collaboration and Innovation in Brain and Learning Sciences, Beijing Normal UniversityBeijing, China

**Keywords:** magneto-acoustical, stimulation, Hodgkin-Huxley model, neuron, parameters

## Abstract

Transcranial magneto-acoustical stimulation (TMAS) is a novel stimulation technology in which an ultrasonic wave within a magnetostatic field generates an electric current in an area of interest in the brain to modulate neuronal activities. As a key part of the neural network, neurons transmit information in the nervous system. However, the effect of TMAS on the neuronal firing pattern remains unknown. To address this problem, we investigated the stimulatory mechanism of TMAS on neurons, by using a Hodgkin-Huxley neuron model. The simulation results indicated that the magnetostatic field intensity and ultrasonic power affect the amplitude and interspike interval of neuronal action potential under a continuous wave ultrasound. The simulation results also showed that the ultrasonic power, duty cycle and repetition frequency can alter the firing pattern of neural action potential under pulsed wave ultrasound. This study may help to reveal and explain the biological mechanism of TMAS and to provide a theoretical basis for TMAS in the treatment or rehabilitation of neuropsychiatric disorders.

## Introduction

Transcranial magnetic stimulation (TMS), a noninvasive brain stimulation tool, has been used for treating and rehabilitating neurological and psychiatric disorders (Hallett, 2000; Golestanirad et al., 2012; Laakso and Hirata, 2012). However, the spatial resolution of TMS is more than several centimeters because the magnetic field cannot be effectively concentrated (Bystritsky et al., 2011). Additionally, the stimulation depth of TMS is limited because the alternating magnetic fields obey Laplace's Equation ∇^2^φ = 0, where φ is the electric potential (Norton, 2003).

Compared with TMS, transcranial focused ultrasound stimulation (tFUS) is a noninvasive method for brain stimulation that can complete a stimulation at a high millimeter-resolution level (< 2 mm; Tufail et al., 2010). More than 40 years ago, Fry et al and Ballantine et al have demonstrated that tFUS had neuromodulatory potential (Fry, 1958; Ballantine et al., 1960). Its effectiveness in neurostimulation has been confirmed in mice, rats, monkeys and humans (Tufail et al., 2010; Deffieux et al., 2013; King et al., 2014; Legon et al., 2014). tFUS can stimulate intact brain circuits of mice and can modulate visuomotor behavior in monkeys as well as the activity of the primary somatosensory cortex in humans.

In this paper, we study the effect of transcranial magneto-acoustical stimulation (TMAS) on the brain. TMAS is a novel brain stimulation technology that was proposed by Norton in 2003 (Norton, 2003). In Norton's paper, this method was analyzed to stimulate nerve tissue, and the calculation results showed that it could be used to locally stimulate active tissue, according to Maxwell's theory. TMAS can generate an electric current in a static magnetic field, using ultrasonic waves to stimulate brain tissues. The spatial resolution of TMAS is determined by the size of the ultrasonic spot, which is approximately 2 mm in diameter. Because the ultrasound and the magnetostatic field strength have good penetration depths, TMAS possesses a high spatial resolution and high penetration for brain stimulation.

The neuron is the basic unit of information transmission in the nervous system. A key neuroscience research issue has been determining how information from external stimuli are encoded and transmitted by neurons (Prescott et al., 2006). Because different neuronal firing patterns carry different stimulation signals, the understanding the firing pattern is very important in research on neural information coding. The mechanism of the neuronal firing pattern and the change of different firing patterns are important to the nervous system. Different external stimuli induce different neuronal firing rhythms and physiological effects. The exploration of the neuronal firing pattern and its underlying mechanisms are of great significance to the study of TMAS and its clinical application.

The relationship between the changes in neuronal firing patterns and TMAS has yet to be determined. Here, we investigated the effect of TMAS on the amplitude (AMP), interspike interval (ISI: the interval of adjacent action potentials per burst) and firing rate (FR: the firing number of the action potential per stimulation cycle) of neuronal action potentials, on the basis of a Hodgkin-Huxley (H-H) neuron model.

## Methods

### Principle of TMAS

The principle of TMAS is that the action of a focused ultrasonic wave moves charged ions in the nerve tissue. Because a magnetostatic field is perpendicular to the movement direction of the charged ions, the Lorentz force on the ions can be induced in the tissue (Ammari et al., 2015; Grasland-Mongrain et al., 2015). The Lorentz force separates the positive and negative ions in opposite directions, thereby forming the electric current *I*_*ext*_ to stimulate neurons.

**Figure 1:**
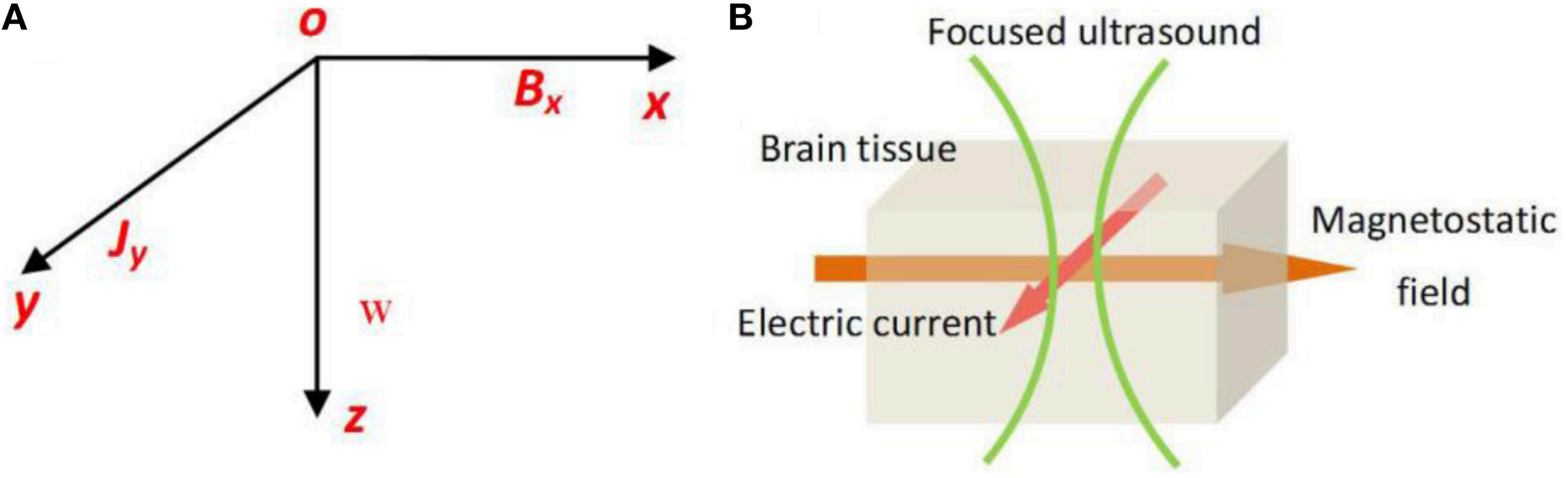
**(A)** The usual Cartensian co-ordinate axes, the pressure waves longitudinal and propagating along *z* axis, and the magetostatic field along *x* axis, the created electric current density along y axis. **(B)** The schematic of TMAS principle.

In our study, standard Cartesian coordinate axes were used. We assumed that the pressure waves were longitudinal and propagated along the *z* axis and that the magnetostatic field was along the *x* axis, thereby placing the current density along the *y* axis (Figures 1A,B).

In the Cartesian coordinate axes, a longitudinal pressure wave propagating along the *z* axis obeys the classical wave Equation

(1) $$\begin{array}{l} \frac{\partial^{2}u}{\partial z^{2}}=\frac{1}{c_{0}^{2}}\frac{\partial^{2}u}{\partial t^{2}} \end{array}$$

where *u* is the distance of the ion from its equilibrium position. In the case of a progressive sine wave, the instantaneous speed *v*_*z*_ of the ion can be expressed as

(2) $$\begin{array}{l} v_{z}=w\text{sin}\left(\omega \text{t}-\varphi \right) \end{array}$$

where *w* is the magnitude of the speed of the ion. ω = *2*π*f* is the angular frequency, where *f* is the ultrasound frequency.

The relationship between the magnitude of the speed of a fluid element and the magnitude of the instantaneous pressure *P* can be expressed as

(3) $$\begin{array}{l} P=\rho {\text{c}}_{0}w \end{array}$$

where ρ is the tissue density and *c*_0_ is the ultrasound speed.

According to Montalibet's theory (Montalibet et al., 2001), the current density *J*_y_ along the y axis generated by ultrasound and magnetostatic fields in a biological medium can be expressed as

(4) $$\begin{array}{l} J_{\text{y}}=\sigma \frac{wB_{x}}{1+{\text{tan}}^{2}\psi }\text{sin}\left(\omega t-\psi \right) \end{array}$$

where σ is the conductivity of the tissue. A typical value of the conductivity of tissue is 0.5 Siemens/m (Norton, 2003). *B*_*x*_ is the intensity of the magnetostatic field. Ψ is the phase angle and obeys the following Equation

(5) $$\begin{array}{l} \text{tan}\psi =\omega \tau \end{array}$$

where τ is the time constant and is on the order of femtoseconds for typical electrolytes. At the ultrasound frequency generally used (200–700 kHz) in ultrasound stimulation (Tufail et al., 2011), the quantities of tanΨ and Ψ are small and negligible with the respect to unity. Therefore, the Equation can be expressed as

(6) $$\begin{array}{l} J_{\text{y}}\approx \sigma wB_{x}\text{sin}\omega \text{t} \end{array}$$

The relationship between the intensity of the ultrasonic power and ultrasound pressure satisfies the following Equation (Hendee and Ritenour, 2002)

(7) $$\begin{array}{l} \Gamma =\frac{1}{2}\frac{P^{2}}{\rho c_{0}} \end{array}$$

where Γ is the intensity of the ultrasonic power.

Combining Equations (6) and (7), we obtained the following Equation

(8) $$\begin{array}{l} J_{\text{y}}\approx \sigma B_{x}\sqrt{\frac{2\Gamma }{\rho c_{0}}}\text{sin}\left(2\pi ft\right) \end{array}$$

If the ultrasonic wave is a cosine wave, the current density can be expressed as

(9) $$\begin{array}{l} J_{\text{y}}\approx \sigma B_{x}\sqrt{\frac{2\Gamma }{\rho c_{0}}}\text{cos}\left(2\pi ft\right) \end{array}$$

The fixed parameters for Equation (8) and (9) are listed in Table 1. The value of electric current density *J*_*y*_, which corresponds to the electric current *I*_*ext*_, can be used to stimulate the neuron and was used for simulation in the H-H neuron model.

**Table 1:** **Fixed parameters for Equation (8) and (9)**.

**Parameters**	**Values**	**Unit**
∑	0.5	Siemens/m
*B*	0.5–7	Teslas
Γ	1–100	Watt/cm^2^
*f*	200–700k	Hz
ρ	1120	Kg/m^3^
*c_0_*	1540	m/s

### H-H neuron model

The H-H neuron model includes the following differential Equations (Hodgkin and Huxley, 1952)

(10) $$\begin{array}{rcl} C_{m}\frac{dV}{dt} & = & I_{ext}-\left[{ḡ}_{Na}m^{3}h\left(V-V_{Na}\right)+{ḡ}_{K}n^{4}\left(V-V_{K}\right)+g_{L}\left(V-V_{L}\right)\right]\\ \frac{dm}{dt} & = & \varphi \left[\alpha_{m}\left(V\right)\left(1-m\right)-\beta_{m}\left(V\right)m\right]\\ \frac{dh}{dt} & = & \varphi \left[\alpha_{h}\left(V\right)\left(1-h\right)-\beta_{h}\left(V\right)h\right]\\ \frac{dn}{dt} & = & \varphi \left[\alpha_{n}\left(V\right)\left(1-n\right)-\beta_{n}\left(V\right)n\right] \end{array}$$

where *C*_*m*_ is the membrane capacitance, and *I*_*ext*_ is the external current generated by the ultrasound and magnetic field in nerve tissue. *V* is the membrane potential and can be expressed as *V* = *V*_*intra*_−*V*_*extra*_ . *V*_*intra*_ and *V*_*extra*_ are the intracellular and extracellular membrane potentials, respectively. φ = 3^(*T*−6.3)∕10^ modifies the time constants of gating variables depending on temperature *T*. *T* = 6.3°C was chosen in the simulation because we did not consider the effect of temperature on the electrical activity of neurons. *m* and *h* are the gating variables representing the activation and inactivation of the Na^+^ current, respectively. *n* is the gating variable representing the activation of the K^+^ current. *V*_*Na*_*, V*_*K*_, and *V*_*L*_ are the equilibrium potentials of sodium, potassium and the leak current, respectively. *g*_*Na*_*, g*_*K*_, and *g*_*L*_ are the maximum conductances of the corresponding ionic currents and are nonlinear functions of *V*, given by the following Equations:

(11) $$\begin{array}{rcl} \alpha_{m}\left(V\right) & = & 0.1\left(25-V\right)∕\left[\text{exp}\left(\left(25-V\right)∕10\right)-1\right]\\ \beta_{m}\left(V\right) & = & 4\text{exp}\left(-V∕18\right)\\ \alpha_{h}\left(V\right) & = & 0.07\text{exp}\left(-V∕20\right)\\ \beta_{h}\left(V\right) & = & 1∕\left[\text{exp}\left(\left(-V+30\right)∕10\right)+1\right]\\ \alpha_{n}\left(V\right) & = & 0.01\left(10-V\right)∕\left[\text{exp}\left(\left(10-V\right)∕10\right)-1\right]\\ \beta_{n}\left(V\right) & = & 0.125\text{exp}\left(-V∕80\right) \end{array}$$

The fixed parameters used for the simulation are listed in Table 2. To make the resting potential equal to zero in the H-H neuron model, the value of the membrane potential was shifted by 65 mV. The initial values of *V, m, h, n* are 0, 0.053, 0.596, and 0.317, respectively. The simulation was performed with Matlab Simulink software (MathWorks, USA).

**Table 2:** **Fixed parameters for Hodgkin-Huxley neuron model**.

**Parameters**	**Values**	**Unit**
*C*_*m*_	1.0	μF/cm^2^
${\bar{g}}_{Na}$	120	mS/cm^2^
${\bar{g}}_{K}$	36	mS/cm^2^
*g*_*L*_	0.3	mS/cm^2^
*V*_*Na*_	115	mV
*V*_*K*_	–12	mV
*V*_*L*_	10.59	mV
*T*	6.3	°C

### The fundamental wave of ultrasound

**Figure 2:**
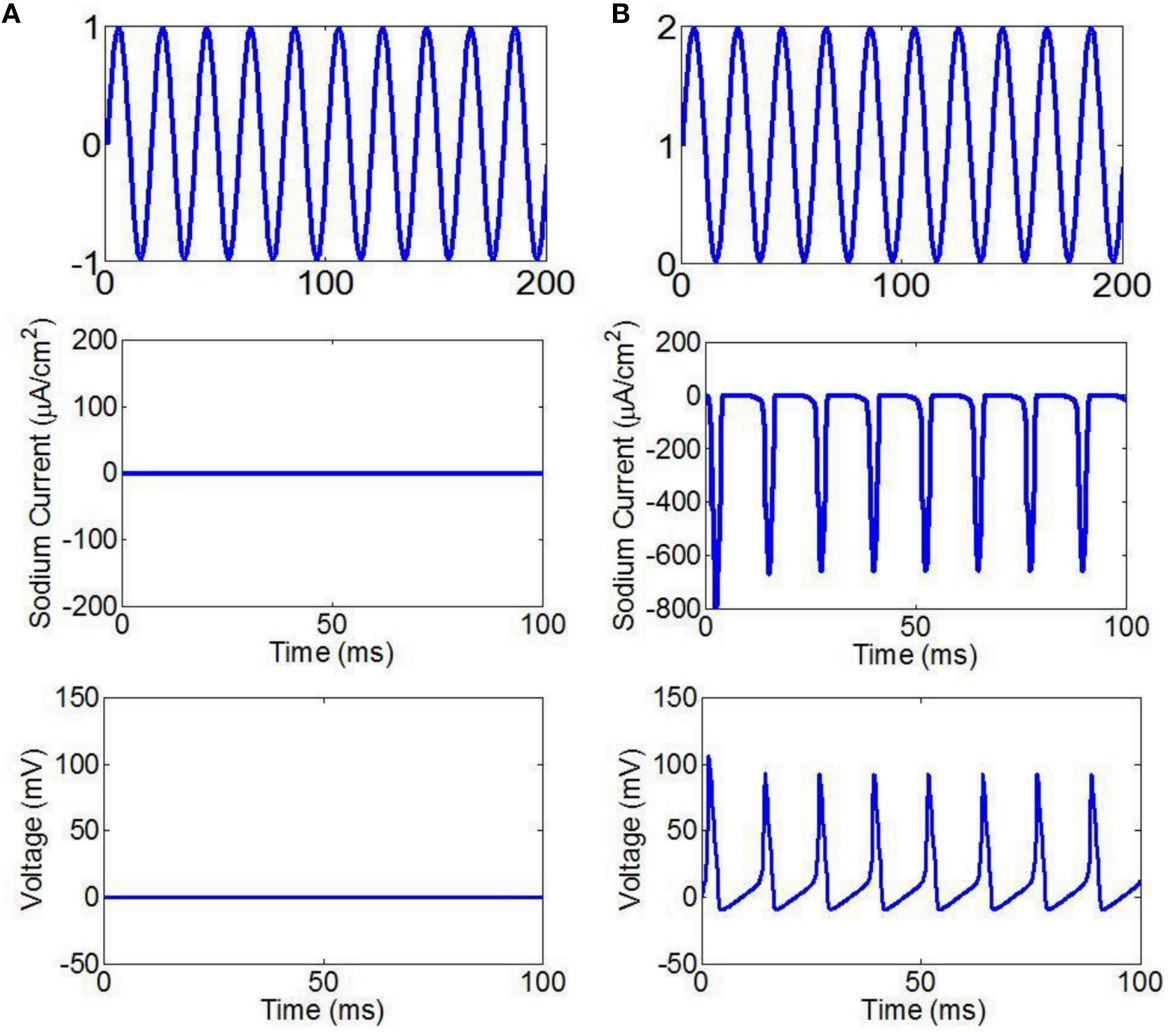
**(A)** The fundamental wave of ultrasound with sine wave (top), the sodium current (center), the neuronal action potentials (bottom). **(B)** The fundamental wave of ultrasound with sine wave with offset (top), the sodium current (center), the neuronal action potentials (bottom).

We assumed that the fundamental wave of ultrasound is a cosine wave. The simulation results are shown in Figure 2. When ultrasound and the magnetic field are under the same intensity, we found that the stimulation cannot make the neurons generate sodium current and action potentials if the fundamental wave of ultrasound is a sine wave (sin(2π*ft*)), (Figure 2A). If the fundamental wave of ultrasound is a sine wave with offset (sin(2π*ft*) + 1), the neurons are able to generate sodium current and action potentials with stimulation (Figure 2B). According to Equation (9), if the fundamental wave of ultrasound is a cosine wave, the electric current changes with the cosine, the sodium channel opens and then closes in a very short period of time ($\frac{10^{-5}}{7}\sim \frac{10^{-5}}{2}s$), and there is not enough time to generate an action potential. If the fundamental wave of ultrasound is a sine wave with offset, there is no negative phase in the electric current, the sodium channel can be opened for a long period of time, and the action potential can be generated.

### Ultrasonic types and parameters

**Figure 3:**
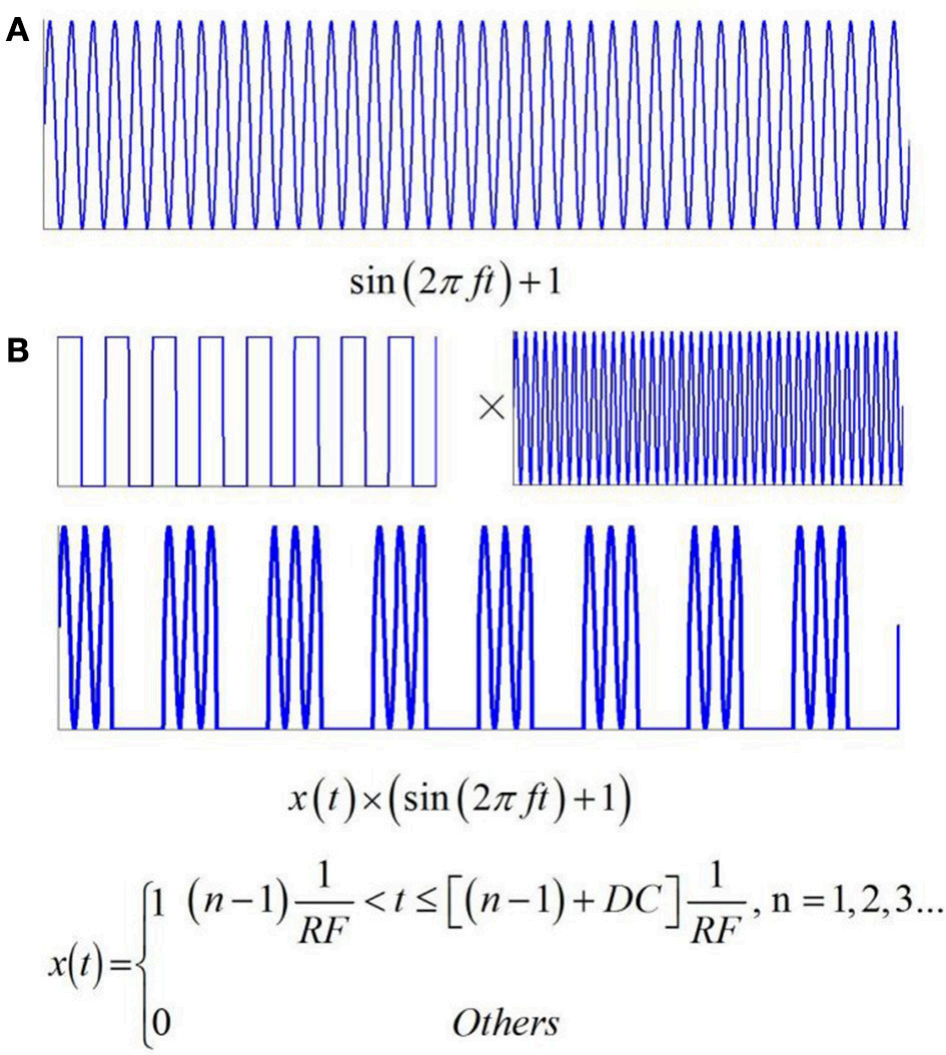
**Two types of ultrasound and the related electric current**. **(A)** Continuous wave ultrasound. **(B)** Pulsed ultrasound with the modulation of square wave and continuous wave.

If the intensity of the magnetostatic field is constant, the current density of the nerve tissue is a function of the ultrasonic power, according to Equation (9). In this study, two types of ultrasound were used in the simulation. The first type, shown in Figure 3A, is continuous wave ultrasound, and the fundamental wave of ultrasound is a sine wave with offset (sin(2π*ft*) + 1). The second type is pulsed ultrasound, with the modulation of a square wave and continuous wave (Figure 3B). It obeys the following Equation:

(12) $$\begin{array}{l} x\left(t\right)=\left\{ \begin{array}{l} 1\left(n-1\right)\frac{1}{RF}<t\leq \left[\left(n-1\right)+DC\right]\frac{1}{RF},n=1,2,3\ldots \\ 0\;Others \end{array}\right. \end{array}$$

where *RF* is the repetition frequency and DC is the duty cycle.

## Results

### Effect of TMAS under continuous wave ultrasound on the firing pattern of neuronal action potential

#### Magnetostatic field intensity

**Figure 4:**
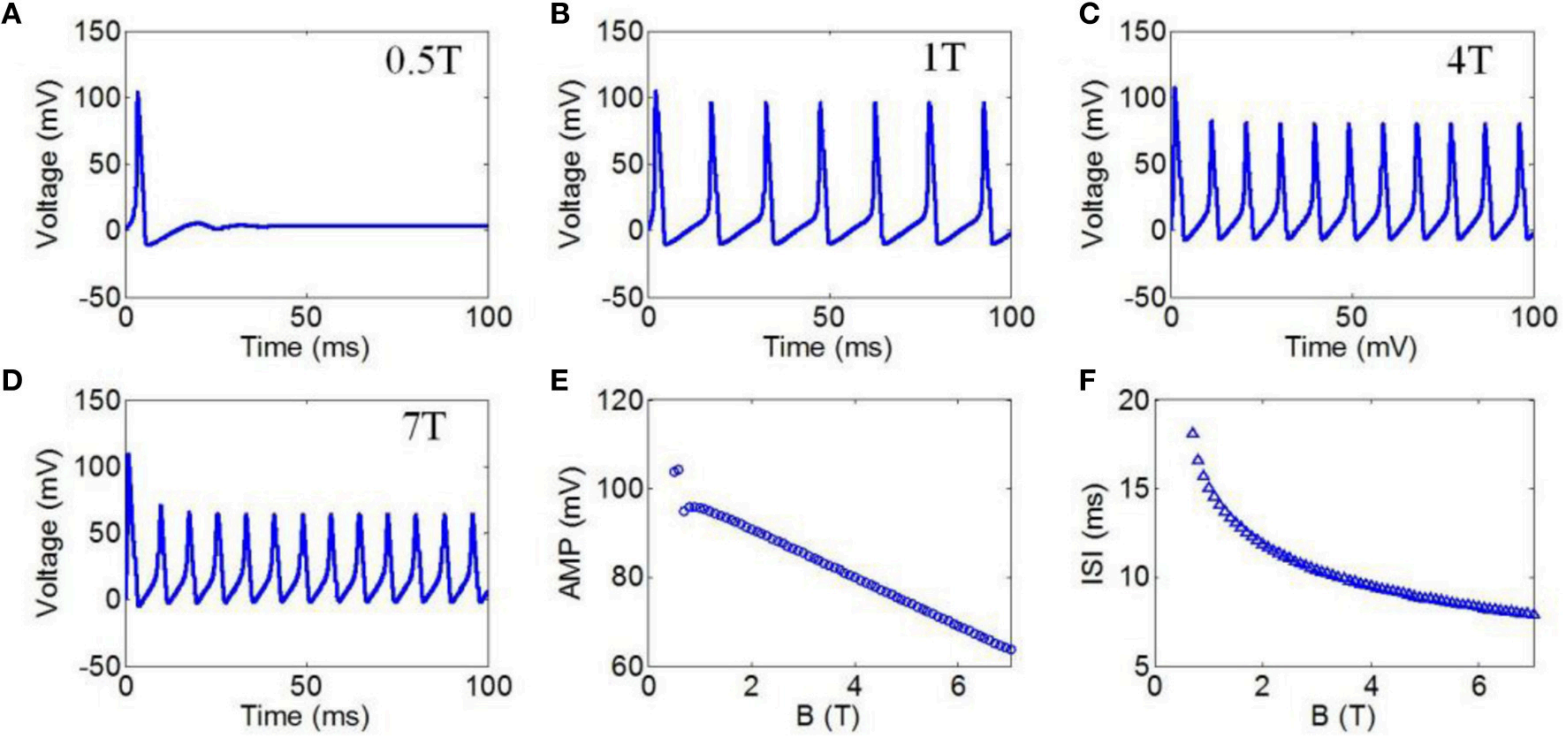
**(A–D)** Waveforms of neuronal action potentials generated by TMAS under continuous wave ultrasound with different magnetostatic field intensity, **(A)** 0.5 T, **(B)** 1 T, **(C)** 4 T, **(D)** 7 T. **(E–F)**. The AMP and ISI of action potentials vs. magnetostatic field intensity, **(E)** AMP, **(F)** ISI.

First, to evaluate the effect of TMAS on the firing pattern of neuronal action potential, the action potential was simulated on the basis of the H-H neuron model under continuous wave ultrasound with various magnetostatic field intensities, from 0.5 to 7 Teslas (T). The values of ultrasonic power and ultrasound frequency were 3 Watt/cm^2^ (W/cm^2^) and 500 kHz, respectively. Figures 4A–D shows the waveforms of action potentials corresponding to magnetostatic field intensities of 0.5, 1, 4, and 7 T, respectively. Additionally, the AMP and ISI of the action potential were calculated to quantitatively analyze the effect of the magnetostatic field potential on the neuronal firing pattern. The AMP of the action potential in relation to the magnetostatic field intensity is shown in Figure 4E. The AMP decreased with the increase in magnetostatic field intensity. Figure 4F shows the ISI of the action potential in relation to ultrasonic power. The results showed that the ISI significantly decreased with increasing magnetostatic field intensity from 0.5 to 7 T.

#### Ultrasonic power

**Figure 5:**
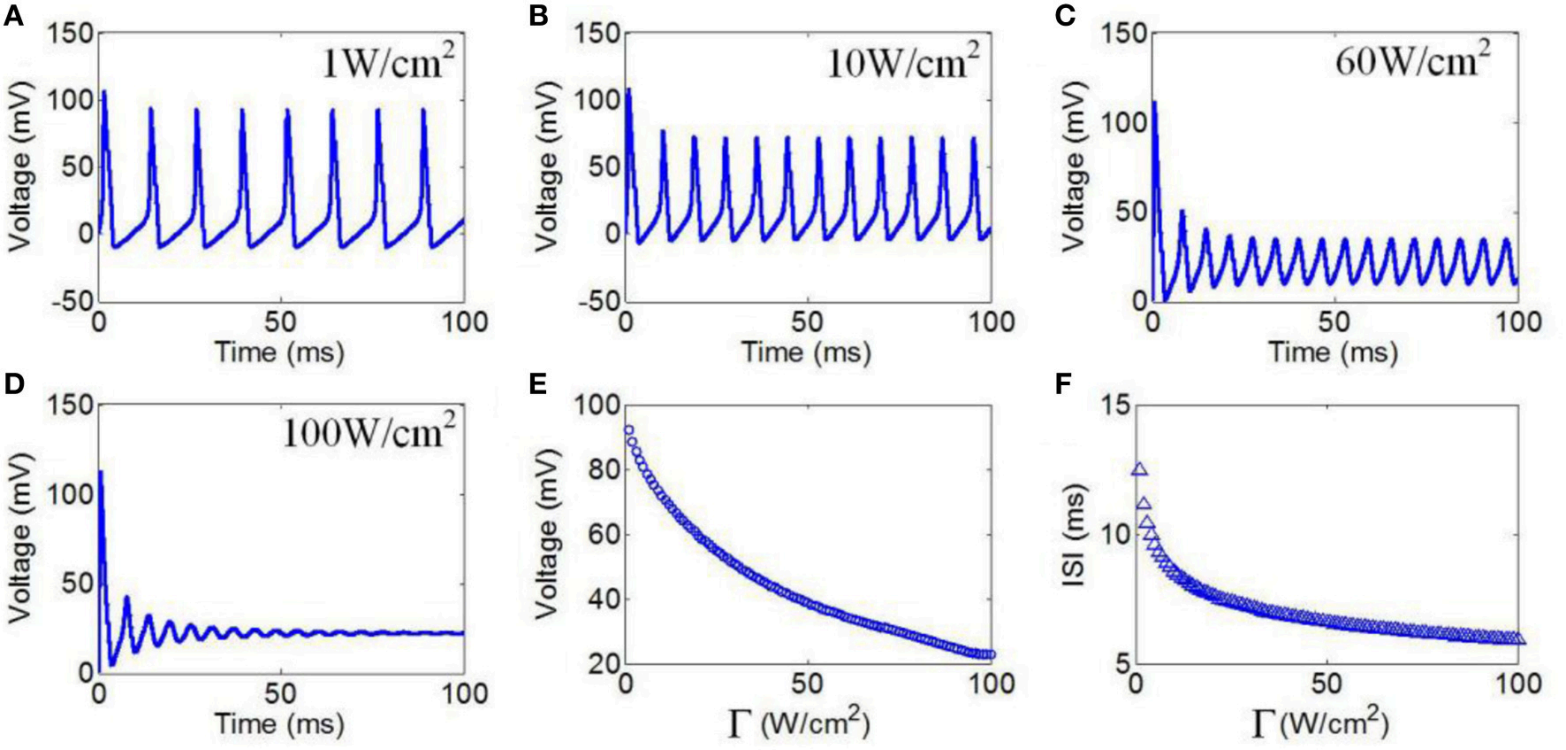
**(A–D)** Waveforms of neuronal action potentials generated by TMAS under continuous wave ultrasound with different ultrasonic powers, **(A)** 1 W/cm^2^, **(B)** 10 W/cm^2^, **(C)** 60 W/cm^2^, **(D)** 100 W/cm^2^. **(E,F)**. The AMP and ISI of action potentials vs. ultrasonic powers, **(E)** AMP, **(F)** ISI.

Next, we evaluated the effect of TMAS on the firing pattern of neuronal action potential under continuous wave ultrasound with various ultrasonic powers. According to the Hopf bifurcation theorem (Wang et al., 2004), if the current density is greater than 9.78 μA/cm^2^ (corresponding to an ultrasonic power of 0.73 W/cm^2^), the neuron can generate periodic action potential. Ultrasonic powers from 1 to 100 W/cm^2^ were used in the simulation to generate action potential. These values were less than 190 W/cm^2^, which is the maximum recommended limit for diagnostic imaging applications (Nyborg, 2001). The values of the magnetostatic field potential and the ultrasound frequency were 3 T and 500 kHz, respectively. Figures 5A–E shows the waveforms of action potentials with ultrasonic powers of 1, 10, 60, and 100 W/cm^2^, respectively. The results showed that there was a decrease in the AMP of the action potential, and the ISI decreased as the ultrasonic power increased. The quantitatively calculated results showed that the AMP significantly decreased with the increase in ultrasonic power (Figure 5E). Figure 5F shows the ISI of the action potentials in relation to ultrasonic power. The results showed a dramatically shortened ISI with increasing ultrasonic powers from 1 to 100 W/cm^2^, after which the narrowing of the ISI range was very limited.

#### Ultrasound frequency

**Figure 6:**
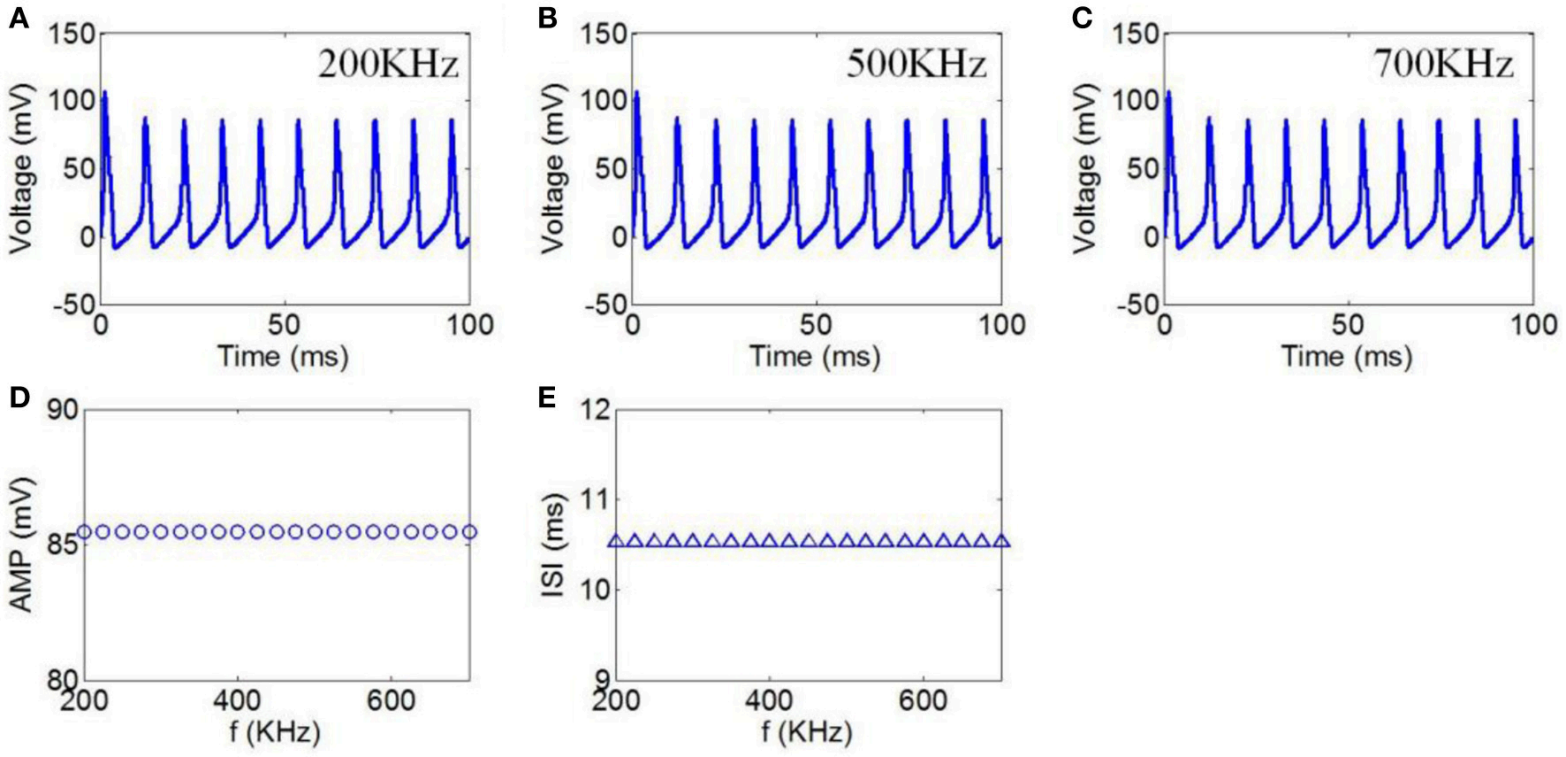
**(A–C)** Waveforms of neuronal action potentials generated by TMAS under continuous wave ultrasound with ultrasound frequency, **(A)** 200 kHz, **(B)** 500 kHz, **(C)** 700 kHz. **(D,E)** The AMP and ISI of action potentials vs. ultrasound frequency, **(D)** AMP, **(E)** ISI.

Finally, we chose ultrasound frequencies from 200 to 700 kHz to evaluate the effect of TMAS on the neuronal firing pattern under continuous wave ultrasound. The magnetostatic field intensity and ultrasonic power were 3 T and 3 W/cm^2^, respectively. The action potentials with ultrasound frequencies of 200, 500, and 700 kHz are shown in Figures 6A–C, respectively. The AMP and ISI of the action potential in relation to ultrasound frequency are shown in Figures 6D,E. The results showed that there were no significant changes in the AMP and ISI of the action potential.

### Effect of TMAS under pulsed ultrasound on the firing pattern of neuronal action potential

#### Ultrasonic power

**Figure 7:**
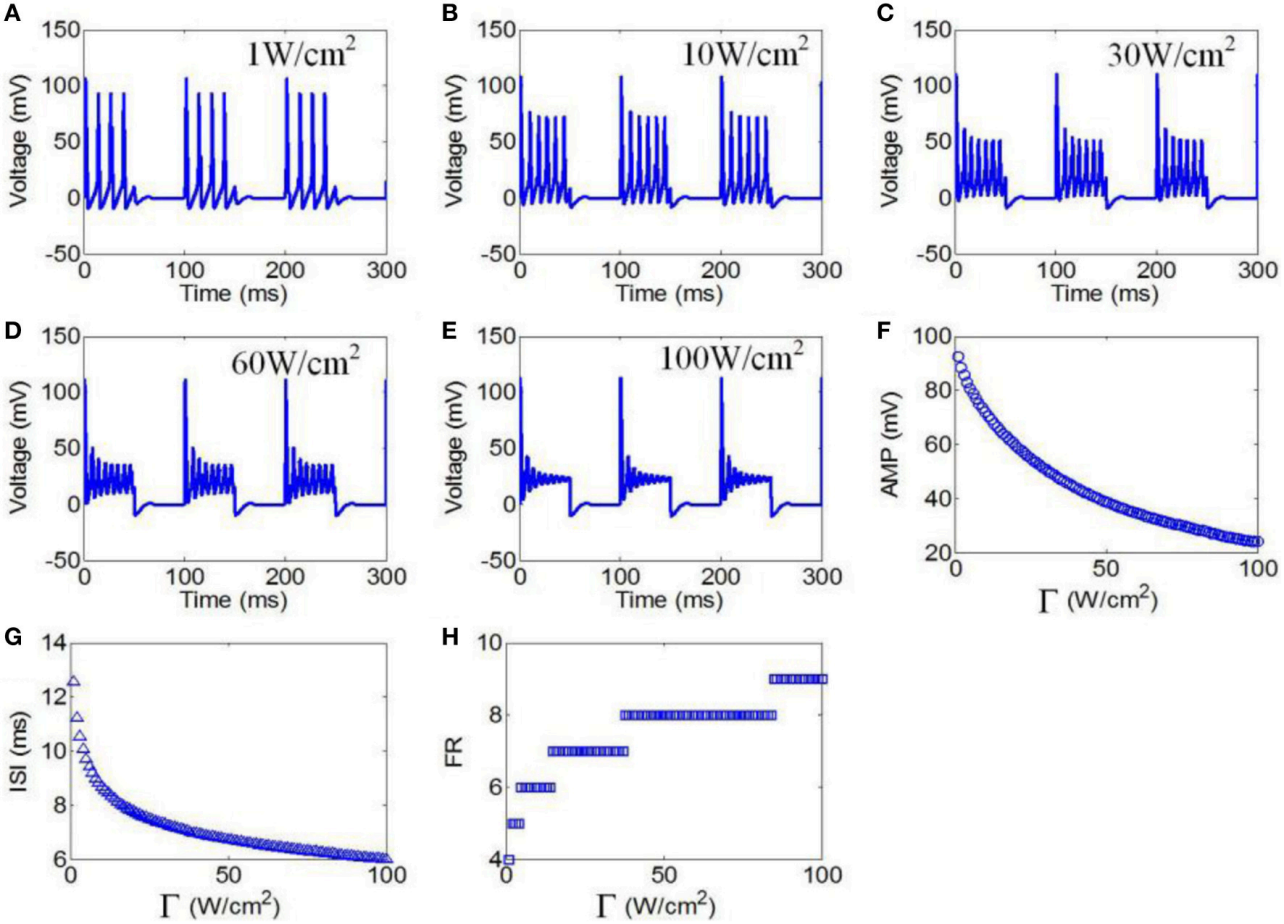
**(A–C)** Waveforms of neuronal action potentials generated by TMAS under pulsed ultrasound with different ultrasound power, **(A)** 1 W/cm^2^, **(B)** 10 W/cm^2^, **(C)** 30 W/cm^2^, **(D)** 60 W/cm^2^, **(E)** 100 W/cm^2^. **(F–H)**. The AMP, ISI and FR of action potentials vs. ultrasonic powers, **(F)** AMP, **(G)** ISI, **(H)** FR.

First, to evaluate the effect of ultrasonic power on the firing pattern of neuronal action potential, the action potential was simulated under pulsed wave ultrasound with various ultrasonic powers. The values of the magnetostatic field strength, ultrasound frequency, duty cycle (DC), and repetition frequency (RF) were 3 T, 500 kHz, 50%, and 10 Hz, respectively. Figures 7A–E shows the waveforms of action potentials with ultrasonic powers of 1, 10, 30, 60, and 100 W/cm^2^, respectively. The neuronal firing form was a cluster discharge with the characteristics of a periodic variation and equal time intervals between the clusters. The number of pulses per cluster increased with the increase in ultrasonic power. The AMP, ISI, and FR of the action potential were quantitatively calculated, and the results are shown in Figures 7F–H. The results showed that the AMP significantly decreased with the increase in ultrasonic power. We also found that the ISI decreased with the increase in ultrasonic power from 1 to 100 W/cm^2^. The FR gradually increased in a multilevel ladder shape. The above results indicate that the firing pattern of neuronal action potential is selective for ultrasonic powers.

#### DC of ultrasound

**Figure 8:**
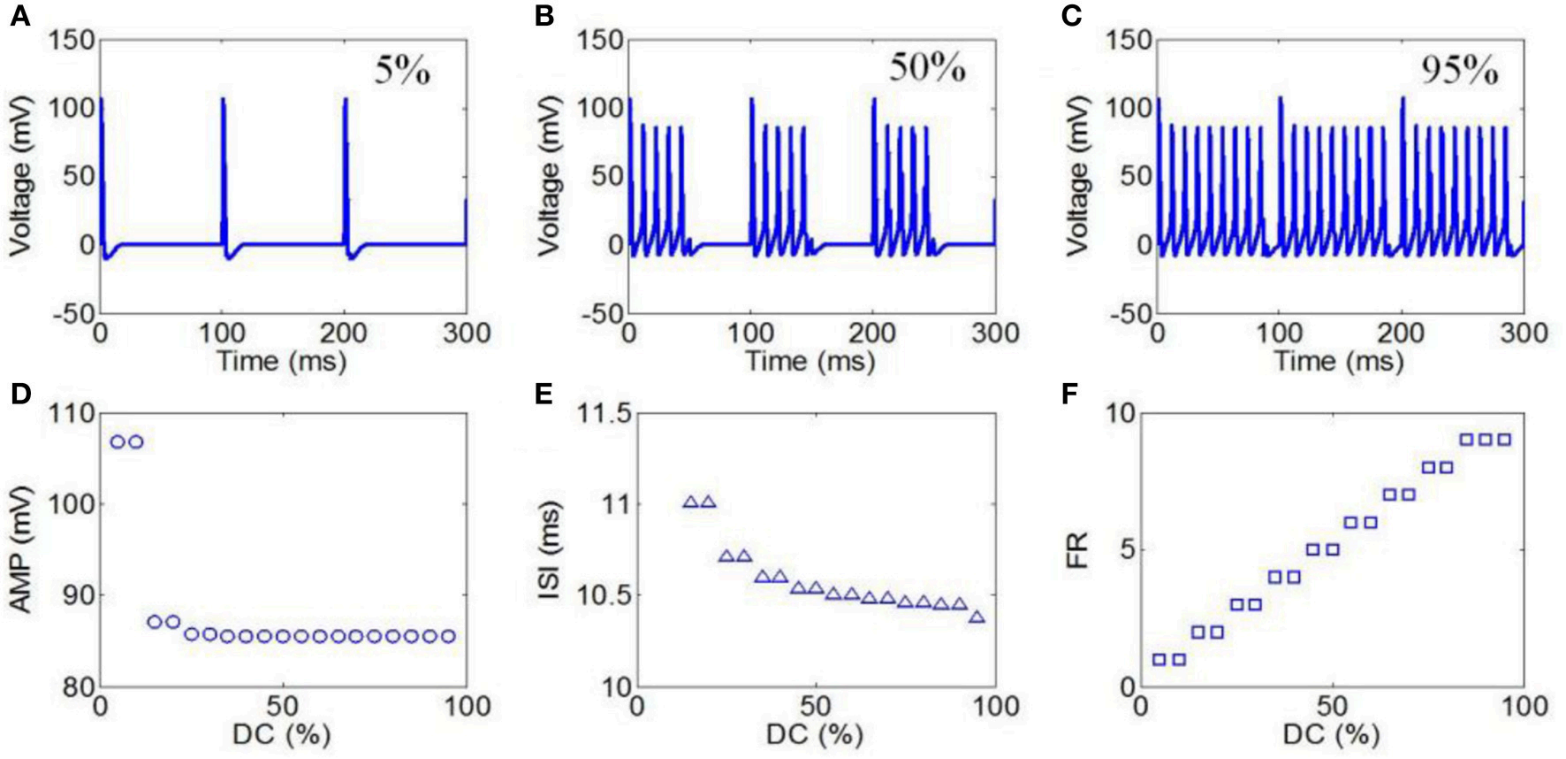
**(A–C)** Waveforms of neuronal action potentials generated by TMAS under pulsed ultrasound with different DC, **(A)** 5 %, **(B)** 50 %, **(C)** 95 %. **(D–F)**. The AMP, ISI, and FR of action potentials vs. DC, **(D)** AMP, **(E)** ISI, **(F)** FR.

Next, DCs from 5 to 95% in intervals of 5% were selected to evaluate the effect of TMAS on the neuronal firing pattern under pulsed ultrasound. The values of the magnetostatic field strength, ultrasound frequency, ultrasonic power, and RF were 3 T, 500 kHz, 3 W/cm^2^, and 10 Hz, respectively. The action potentials with DCs of 5, 50, and 95% are shown in Figures 8A–C, respectively. The firing form of the neuron was also a cluster discharge. The time interval between the clusters decreased with the increase in repetition frequency. The AMP, ISI, and FR of the action potential in relation to the duty cycle are shown in Figures 8D–F, respectively. The results showed a weak decrease in the AMP and ISI with the increase in DC. We also found that the FR gradually decreased with the increase in DC.

#### RF of ultrasound

**Figure 9:**
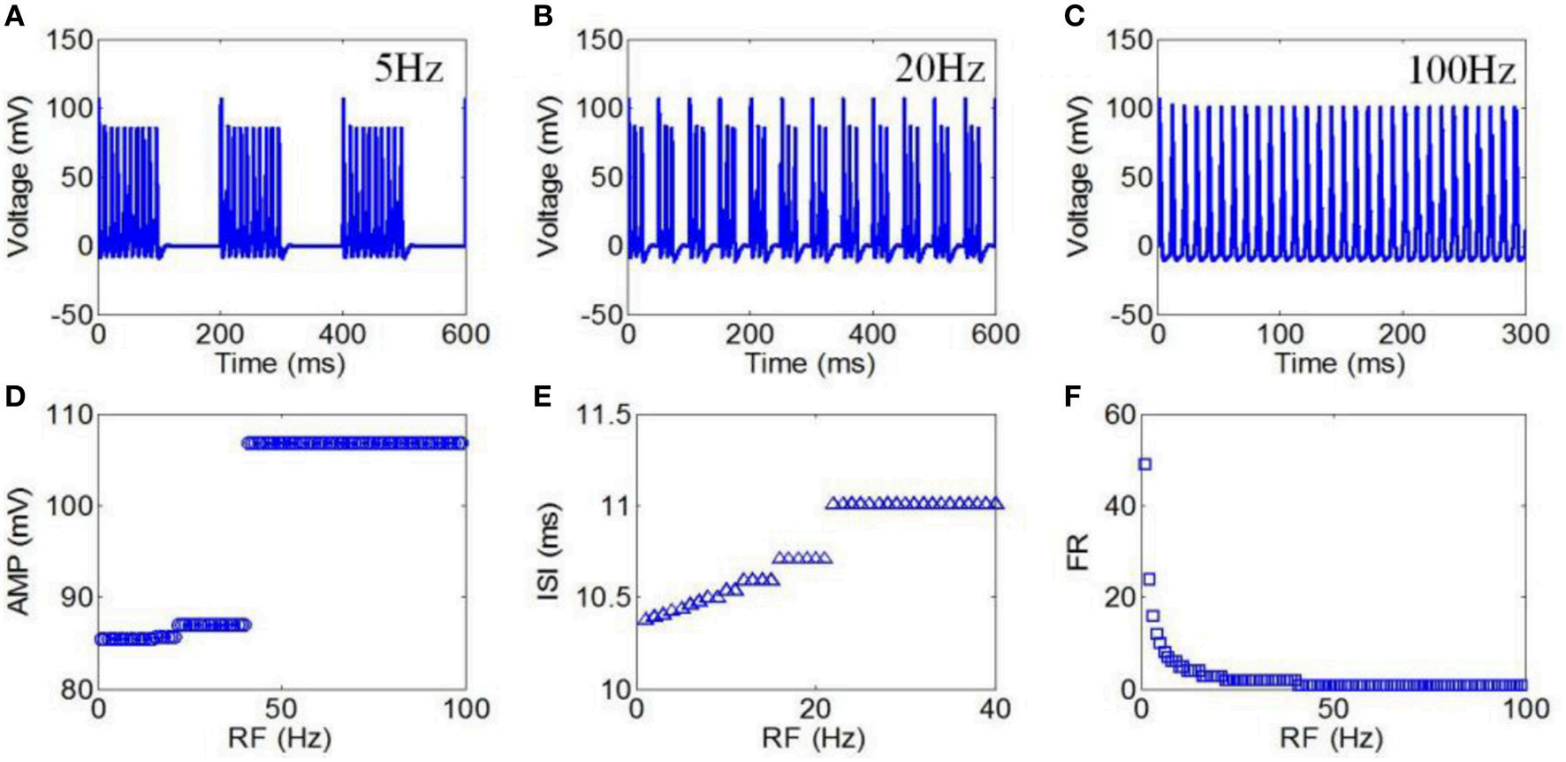
**(A–C)** Waveforms of neuronal action potentials generated by TMAS under pulsed ultrasound with different RF, **(A)** 5 Hz, **(B)** 20 Hz, **(C)** 100 Hz. **(D–F)**. The AMP, ISI, and FR of action potentials vs. RF, **(D)** AMP, **(E)** ISI, **(F)** FR.

Finally, we used RFs from 1 to 100 Hz to evaluate the effect of TMAS on the neuronal firing pattern under pulsed ultrasound. The magnetostatic field strength, ultrasound frequency, ultrasonic power and DC were 3 T, 500 kHz, 3 W/cm^2^, and 50%, respectively. The action potentials with repetition frequencies of 5, 20, and 100 Hz are shown in Figures 9A–C, respectively. The AMP, ISI, and FR of the action potential compared to repetition frequency are shown in Figures 9D–F, respectively. The results showed that both the AMP and ISI gradually decreased in a multilevel ladder shape route with an increase in repetition frequency. We also clearly observed that the FR significantly decreased as the RF increased from 1 to 100 Hz, after which the narrowing of the FR range was very limited.

## Discussion and conclusion

In this study, we investigated the effect of TMAS on the neuronal firing pattern. Our results showed that neurons can generate a rich firing pattern with TMAS. The simulation results showed that neurons have a high sensitivity to changes in ultrasound parameters in TMAS. Small changes in ultrasound parameters can lead to changes in the neuronal firing pattern, thus affecting the neural information coding. These findings lay the foundation for further studies on the effects of TMAS on the neural network and reveal the underlying mechanisms of TMAS in the biological neural system.

Regarding the mechanisms of tFUS and TMAS, a previous study has shown that the mechanism of tFUS is that ultrasound-induced cavitation of these nanometric bilayer sonophores can induce a complex mechanoelectrical interplay that leads to excitation, primarily through the effect of currents induced by membrane capacitance changes (Plaksin et al., 2014). The mechanism of TMAS is that an electric current is generated by the static magnetic field and ultrasonic waves in nerve tissues to modulate neuronal activity. Therefore, the mechanisms of ultrasound stimulation and magneto-acoustical stimulation are different.

Regarding the effect of the parameters of tFUS and TMAS on stimulation results, King et al have studied the effect of different types of stimuli (continuous-wave stimuli and pulsed stimuli) and ultrasonic parameters (acoustic intensity, sonication duration, pulse repetition frequency, etc.) on the stimulation success of ultrasound-induced *in vivo* neurostimulation (King et al., 2014). In our study, we analyzed the effects of magnetic intensity and types of ultrasound (continuous and pulsed ultrasound) with various ultrasonic parameters on the neuronal firing pattern. The simulation results showed that the type of ultrasound, the magnetic intensity, and the ultrasonic parameters induce different neuronal firing patterns. Our results may provide important theoretical guidance for the observation of neuron firing activity in future experiments.

The mechanical index (MI) is the value of the related biological mechanical effects and can be used to estimate the potential biomechanical effects. The thermal index (TI) is used to indicate the temperature rise of the tissue caused by the absorption effect of the ultrasonic energy. The MI and TI formulas presented in the body of the “Standard for real-time display of thermal and mechanical acoustic output indices on diagnostic ultrasound equipment” (Abbott, 1999) are very important for evaluating the MI and TI for TMAS. According to IEC 60601-2-37 standard regulation, the MI can be expressed as $MI=\frac{p_{r.3}}{\sqrt{f}C_{MI}}$, where *C*_*MI*_ equals 1 MPa·MHz^1∕2^, *p*_*r*.3_ is the attenuated peak-rarefactional pressure in MPa, and f is the acoustic working frequency in MHz. The TI for unscanned modes can be expressed as $TI=\frac{Wf}{C_{TIS}}$, where C_*TIS*_ is 210 mW·MHz. In a previous study, an ultrasonic power of 3 W/cm^2^ has been used for ultrasound brain modulation in humans. In our study, the simulation results showed that neurons can also generate an action potential with an ultrasonic power of 3 W/cm^2^, an ultrasound frequency of 0.5 MHz and a magnetic intensity of 3 T. If the ultrasonic power is 3 W/cm^2^, the ultrasound frequency is 0.5 MHz, and the diameter of the ultrasonic spot is 3 mm. With a *p*_*r*.3_ of 0.2275 MPa, an ultrasound spot area of 7.065 × 10^−2^ cm^2^ and a *W* of 212 mW, we obtained an MI of 0.32 and a TI of 0.505, which were less than the MI and TI threshold (equal 1) that can induce any tissue injury.

Previous studies have shown that abnormal neuronal firing patterns cause some diseases of the nervous system (Valdez et al., 2013). The pathogenesis of epilepsy is excessive synchronization of brain neuronal discharge, resulting in a brain function imbalance (Ding et al., 2006). Our study shows that TMAS can alter the neural firing pattern, thus indicating that it has great potential to intervene in an epileptic seizure. We also found that the ultrasonic parameters can affect the neuronal firing pattern; hence, we can change these parameters during the treatment of epileptic seizures to achieve an optimal therapeutic effect.

In summary, the current investigation demonstrates that TMAS can alter the neuronal firing pattern in two ways: (i) the magnetostatic field intensity and ultrasonic power are able to affect the AMP and ISI of neuronal action potential under continuous wave ultrasound, and (ii) the ultrasonic power, DC and RF can alter the firing pattern of neural action potential under pulsed ultrasound. Our results suggest that TMAS has potential as a powerful noninvasive method to interfere with brain rhythms for diagnostic and therapeutic purposes.

## Author contributions

YY and XL designed and coordinated the study, YY and YC carried out numerical implementation of the TMAS, done the simulation and drafted the manuscript. All authors gave final approval for publication.

### Conflict of interest statement

The authors declare that the research was conducted in the absence of any commercial or financial relationships that could be construed as a potential conflict of interest.

## Abbreviations

TMAS: Transcranial magneto-acoustical stimulation
H-H: Hodgkin-Huxley
TMS: Transcranial magnetic stimulation
tFUS: Transcranial focused ultrasound stimulation
AMP: amplitude
ISI: interspike internal
FR: firing rate
DC: duty cycle
RF: repetition frequency
MI: mechanical index
TI: thermal index.
